# Regulation by SoxR of *mfsA*, Which Encodes a Major Facilitator Protein Involved in Paraquat Resistance in *Stenotrophomonas maltophilia*

**DOI:** 10.1371/journal.pone.0123699

**Published:** 2015-04-27

**Authors:** Kriangsuk Srijaruskul, Nisanart Charoenlap, Poommaree Namchaiw, Sorayut Chattrakarn, Suparat Giengkam, Skorn Mongkolsuk, Paiboon Vattanaviboon

**Affiliations:** 1 Laboratory of Biotechnology, Chulabhorn Research Institute, Bangkok, 10210, Thailand; 2 Program in Applied Biological Sciences: Environmental Health, Chulabhorn Graduate Institute, Bangkok, 10210, Thailand; 3 Department of Biotechnology, Faculty of Science, Mahidol University, Bangkok, 10400, Thailand; 4 Center of Emerging Bacterial Infection, Faculty of Science, Mahidol University, Bangkok, 10400, Thailand; 5 Center of Excellence on Environmental Health and Toxicology, Bangkok, Thailand; Arizona State University, UNITED STATES

## Abstract

*Stenotrophomonas maltophilia* MfsA (Smlt1083) is an efflux pump in the major facilitator superfamily (MFS). Deletion of *mfsA* renders the strain more susceptible to paraquat, but no alteration in the susceptibility levels of other oxidants is observed. The expression of *mfsA* is inducible upon challenge with redox cycling/superoxide-generating drug (paraquat, menadione and plumbagin) treatments and is directly regulated by SoxR, which is a transcription regulator and sensor of superoxide-generating agents. Analysis of *mfsA* expression patterns in wild-type and a *soxR* mutant suggests that oxidized SoxR functions as a transcription activator of the gene. *soxR* (smlt1084) is located in a head-to-head fashion with *mfsA*, and these genes share the -10 motif of their promoter sequences. Purified SoxR specifically binds to the putative *mfsA* promoter motifs that contain a region that is highly homologous to the consensus SoxR binding site, and mutation of the SoxR binding site abolishes binding of purified SoxR protein. The SoxR box is located between the putative -35 and -10 promoter motifs of *mfsA*; and this position is typical for a promoter in which SoxR acts as a transcriptional activator. At the *soxR* promoter, the SoxR binding site covers the transcription start site of the *soxR* transcript; thus, binding of SoxR auto-represses its own transcription. Taken together, our results reveal for the first time that *mfsA* is a novel member of the SoxR regulon and that SoxR binds and directly regulates its expression.

## Introduction

*Stenotrophomonas maltophilia* is an aerobic, Gram-negative, opportunistic pathogen that is ubiquitous in the environment. This microorganism is of great interest because it is considered to be an emerging nosocomial pathogen with intrinsic antimicrobial resistance to a variety of antibiotics. *S*. *maltophilia* is reported to be associated with infections of cystic fibrosis patients and immunocompromised individuals, particularly those who have been admitted in hospitals for a long period. This pathogen causes meningitis, endocarditis, bacteremia and infections of the respiratory, urinary and gastrointestinal tracts. Blood stream infection of *S*. *maltophilia* usually results in a substantially high mortality rate [1,2].

The major facilitator superfamily (MFS) consists of membrane transport proteins that are attributed to active efflux of compounds, including antibiotics, toxins and xenobiotics through a cation/substrate antiport mechanism [3]. The prokaryotic MFS encompass at least 58 families [4], and MFS efflux pumps contribute to multidrug resistance in several Gram-positive and Gram-negative pathogenic bacteria [5,6,7,8]. The most well-characterized MFS in term of function and gene regulation is a tetracycline efflux protein (TetA) [9,10,11]. The expression of *tetA* is regulated by the TetR transcription repressor. In the absence of tetracycline, TetR binds the *tetA* operator and represses its transcript. Once exposed to tetracycline, the drug-Mg^2+^ complex binds the TetR repressor and changes the conformation of TetR, leading to a release of TetR from the *tetA* operator, and thereby allowing the transcription process of *tetA*.

SoxR, which is a MerR-type transcription factor, is a [2Fe-2S] cluster-containing transcriptional regulator that senses and responds to superoxide stress, redox cycling drugs/superoxide generators and reactive nitrogen species through one-electron oxidation of its [2Fe-2S]^1+^ reactive center to [2Fe-2S]^2+^ [12,13]. In *E*. *coli*, the SoxR homodimer binds to a dyad-symmetric sequence that is located between the -35 and -10 elements of the *soxS* promoter. Under normal physiological conditions, binding of reduced SoxR represses *soxS* transcription, while, under redox stress, binding of oxidized SoxR activates *soxS* gene expression [14]. The synthesized SoxS then up-regulates other genes in the regulon that are involved in stress alleviation and damage repair. While SoxR is highly conserved in diverse bacteria, SoxS is absent from most non-enteric bacteria, and SoxR directly regulates genes in the regulon [15,16,17,18,19,20,21].

Several drug resistance determinants in *S*. *maltophilia* have been investigated and reported, but, to our knowledge, none belong to the MFS family. Here, we report the contribution of *mfsA*, which encodes a MFS protein, to the drug resistance of *S*. *maltophilia*. The transcription of *mfsA* is controlled by SoxR, which is a redox cycling drug/superoxide sensor and transcriptional regulator. Redox cycling drugs such as paraquat, plumbagin and menadione are compounds capable of undergoing enzymatic one-electron reduction to form a transient radical that can be reoxidized by molecular oxygen (O_2_) to produce superoxide anion [22]. Thus, *mfsA* expression is increased in response to the presence of redox cycling drugs. Because *mfsA* and *soxR* have overlapping promoter sequences, binding of SoxR to a single SoxR binding site controls the gene expression of *mfsA* and *soxR*, but in different manners.

## Results and Discussion

### MfsA belongs to a 14 transmembrane domain subfamily of the major facilitator superfamily

At least 37 putative MFS are identified in the annotated genomes of *S*. *maltophilia* [23,24]. Among these coding DNA sequences (CDS), Smlt1083 (hereafter referred to as *mfsA*) is of particular interest because it is located next to a divergent gene, *smlt1084*, encoding a SoxR homolog, which is a superoxide sensor and transcriptional regulator. A linkage between *mfs* and *soxR* has been previously reported in *Xanthomonas campestris* pv. campestris [18]. Analysis of the available bacterial genomes unveiled that the *mfs* and *soxR* gene organization is found in some proteobacteria (including *Xanthomonas axonopodis* and *Agrobacterium vitis*) and actinobacteria (including *Gordonia bronchialis*, *Mycobacterium abscessus*, *Rhodococcus erythropolis*, *Rhodococcus opacus*, *Renibacterium salmoninarum* and *Nocardia farcinica*). Thus far, no functional connection between *mfs* and *soxR* has been reported.

**Fig 1:**
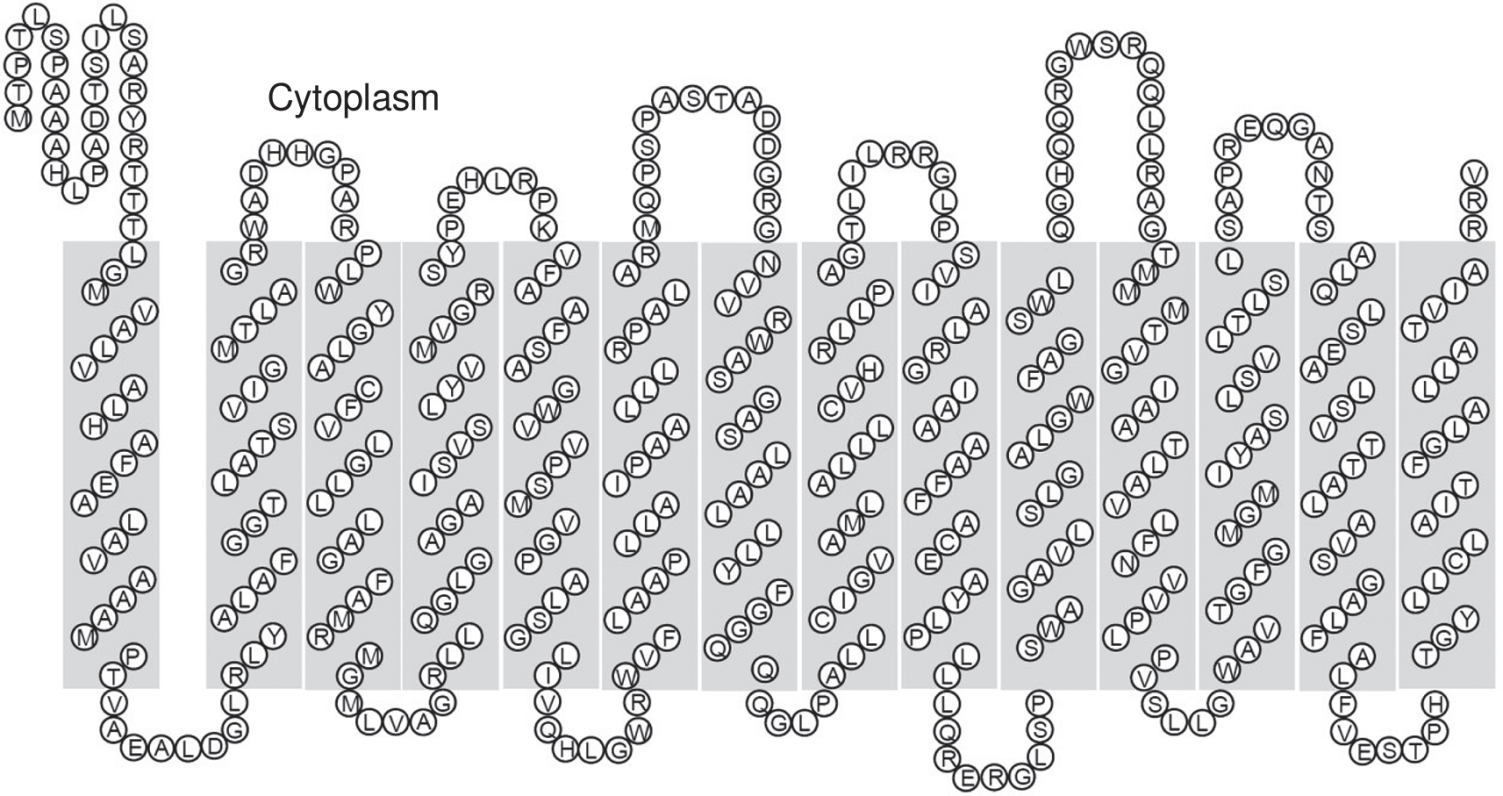
Two-dimensional model of MfsA. The secondary structure of MfsA was predicted using the SOSUI algorithm [43].

The putative *mfsA* encodes a 473-amino-acid protein [23,24]. Our primer extension results (in the promoter analysis section) strongly suggest that a starting codon (ATG) should be located 51 bp downstream of the original ATG that is annotated in the genome sequence database. Thus, *mfsA* encodes a 456-amino-acid protein with a theoretical molecular mass of 49.4 kDa. Analysis of its deduced amino acid sequence using several algorithms for protein topology prediction, viz. TMPred, TMHMM 2.0, SOSUI and TopPred II, strongly suggest that *S*. *maltophilia* MfsA possesses 14 helical transmembrane domains (L28-T50, R59-R81, P91-G113, R119-Y141, V149-I171, W179-R201, N216-Q237, A242-G264, S274-L296, S306-L325, T343-V365, W370-L391, A403-L425 and T433-A453) and exposes its N- and C-termini to the cytoplasm (Fig 1). Generally, transmembrane transport through a MFS is energized by the electrochemical gradient of protons in an antiport process [25]. Most prokaryotic MFS proteins typically contain either 12 or, to a lesser extent, 14 transmembrane α-helical domains [4]. Based on the classification of MFS proteins that was proposed by Pao et al. [26] and phylogenic tree analysis, *S*. *maltophilia* MfsA is suggested to be an efflux pump that belongs to a subfamily of the drug:H^+^ antiporter with 14 transmembrane domain subfamily (DHA14). The representative example of this group is the drug efflux pump QacA, which is an MFS that mediates resistance to antiseptics and disinfectants of Gram-positive *Staphylococcus aureus* [27]. However, the MfsA primary structure shares only 17.0% identity with *S*. *aureus* QacA and 16.1 and 14.4% identity with the methyl viologen resistance protein, SmvA, from *Salmonella enterica* serovar Typhimurium [28] and *Acinetobacter baumannii* [29], respectively. Furthermore, individual members of the MFS subfamily commonly share low amino acid sequence identity and similarity [4].

### The *mfsA* mutant showed increased susceptibility to paraquat

**Fig 2:**
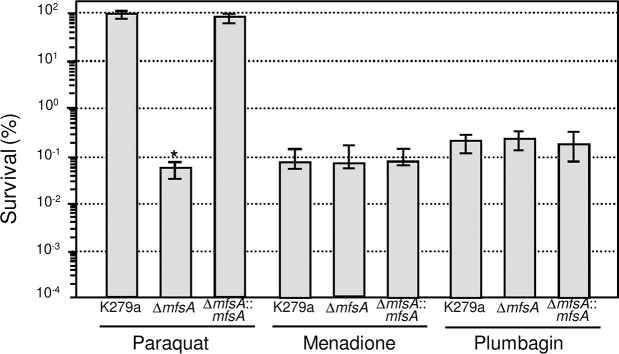
The resistance levels of the *S*. *maltophilia* Δ*mfsA* mutant toward redox cycling drugs. A plate sensitivity assay was conducted to determine the resistance levels to oxidants. Ten-fold serially diluted, exponential-phase *S*. *maltophilia* K279a wild-type, Δ*mfsA* mutant and complemented (Δ*mfsA*::mfsA) cells were spotted onto plates containing 400 μM paraquat, 3 mM menadione or 400 μM plumbagin. The percent survival was defined as the percentage of CFUs on plates containing oxidant over the CFUs on control plates without oxidant.

To evaluate the function of *mfsA* in *S*. *maltophilia*, the Δ*mfsA* mutant was constructed by gene deletion using double crossover and the Cre-*loxP* system [30]. We determined the susceptibility of the Δ*mfsA* mutant against methyl viologen (paraquat), and, as illustrated in Fig 2, the Δ*mfsA* mutant showed increased sensitivity to paraquat. The mutant was 10^3^-fold more susceptible to paraquat when cells were grown in the presence of 400 μM paraquat compared with the wild-type control. The altered phenotype of the Δ*mfsA* mutant was fully restored in the complemented strain (the Δ*mfsA* mutant containing a transposed mini-Tn7-*mfsA* for the single-copy expression of *mfsA* (Δ*mfsA*::*msrA*)). Because paraquat is a redox cycling drug that generates intracellular superoxide anions in the presence of O_2_, we tested whether increased paraquat susceptibility was due to lowering of superoxide dismutase (SOD) activities, as SOD is a group of enzymes that is responsible for dismutation of superoxide anions to H_2_O_2_. Total SOD activity was monitored as previously described [18], and the results showed that SOD activity in the Δ*mfsA* mutant (98.8 ± 7.4 U mg^-1^ protein) was comparable to that of wild-type K279a (95.5 ± 6.3 U mg^-1^ protein) and the *mfsA* complemented strain (96.5 ± 8.0 U mg^-1^ protein). Thus, increased susceptibility of the Δ*mfsA* mutant to paraquat is not due to alteration of total SOD activity.

Next, the sensitivity levels of the Δ*mfsA* mutant against other redox cycling drugs, i.e., menadione and plumbagin, were determined. Fig 2 illustrates that no significant changes in the sensitivity levels against menadione and plumbagin were observed in the Δ*mfsA* mutant relative to wild-type K279a. The fact that the phenotype of the Δ*mfsA* mutant that was sensitive to redox cycling drugs was specific to paraquat indicates that MfsA-mediated protection against paraquat toxicity does not involve superoxide anions. Together with its topology analysis, we proposed that MfsA functions as an efflux pump for paraquat. MfsA shares relatively low identity (14–16%) with the previously characterized paraquat resistance protein SmvA [28,29] in other Gram-negative bacteria. Thus, MfsA is likely a new paraquat resistance MFS protein. Indeed, *S*. *maltophilia* possesses an open reading frame, Smlt2678, that shares 33.9% identity with SmvA from *Salmonella* Typhimurium [28]. The contribution of Smlt2678 to paraquat resistance warrants further investigations.

Experiments were extended to test if deletion of *mfsA* affected the sensitivity levels to other oxidants, such as a disinfectant, H_2_O_2_, or organic hydroperoxides. The results revealed no significant differences in the sensitivity levels to H_2_O_2_, cumene hydroperoxide or *t*-butyl hydroperoxide in the Δ*mfsA* mutant relative to wild-type K279a (data not shown). MfsA clearly has a major role in paraquat resistance, but not in general oxidative stress protection of *S*. *maltophilia*.

### The expression of *mfsA* is induced upon challenge with redox cycling drugs

**Fig 3:**
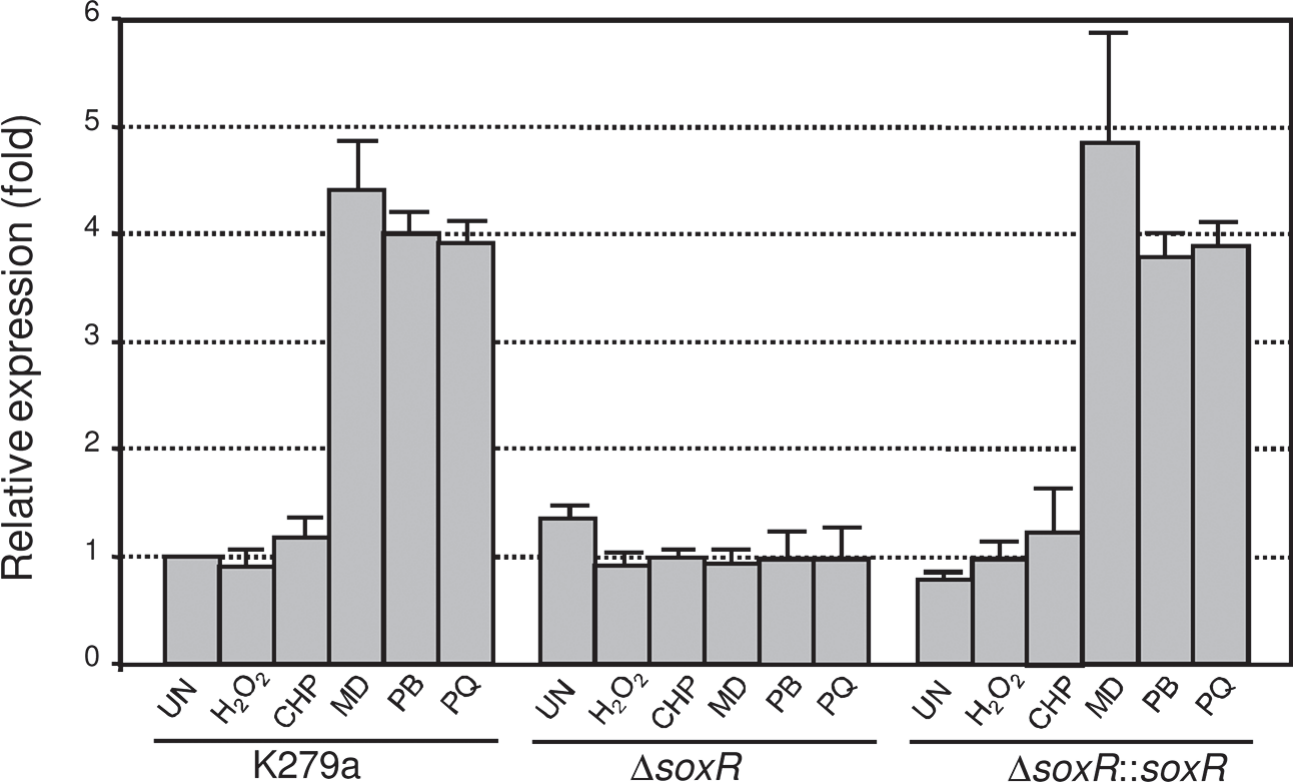
Expression analysis of *mfsA*. The expression levels of *mfsA* in *S*. *maltophilia* K279a wild-type, Δ*soxR* mutant and complemented strain (Δ*soxR*::*soxR*) grown under uninduced (UN) conditions and induced with 100 μM H_2_O_2_, 50 μM cumene hydroperoxide (CHP), 200 μM menadione (MD), 100 μM plumbagin (PB) or 100 μM paraquat (PQ) were determined using qRT-PCR. The 16S rRNA gene was used as the normalizing gene. Relative expression was expressed as the fold expression over the level in uninduced K279a.

Real**-**time reverse transcription polymerase chain reaction (qRT**-**PCR) was performed to measure the expression of *mfsA* in response to stresses. As illustrated in Fig 3, exposure to sublethal concentrations of redox cycling drugs, i.e., menadione, paraquat and plumbagin, enhanced the *mfsA* transcriptional level by 4.4 ± 0.5, 4.0 ± 0.2 and 3.9 ± 0.2-fold, respectively. Challenging the bacteria with H_2_O_2_ or organic hydroperoxides did not induce *mfsA* transcription (Fig 3). The pattern of *mfsA* gene expression clearly shows that superoxide generators/redox cycling drugs are potent inducers of gene expression; and this result couples with the gene organization of *mfsA*, which is located immediately upstream and in the reverse orientation to *soxR* (*smlt1984*), and strongly suggests a contribution of SoxR in the regulation *mfsA* expression.

### SoxR regulates *mfsA* gene expression

SoxR can either repress or activate transcription of its target gene depending on the redox status of the protein. In many bacteria, SoxR functions as a sensor for superoxide anions/redox cycling drugs or a transcription regulator. Based on phylogenetic tree analysis in a recent report, *S*. *maltophila* SoxR is evolutionarily closely related to SoxR from *Xanthomonas campestris* (with 70.1% identity) [31] and shares 63.8, 61.8 and 59.7% identity with SoxR from *Pseudomonas aeruginosa*, *Streptomyces coelicolor* and *E*. *coli*, respectively. The four cysteine residues that anchor the [2Fe-2S] cluster are conserved (data not shown).

To test whether SoxR regulates the expression of *mfsA*, the Δ*soxR* mutant was constructed as described in the Methods and its expression profile was determined. Fig 3 illustrates that the basal level of the *mfsA* transcript in the Δ*soxR* mutant increased moderately (1.3 ± 0.1-fold) relative to the wild-type level. Moreover, expression of *soxR* in the complemented strain (Δ*soxR*::*soxR*), in which a single copy of *soxR* was transposed into the mutant using a mini-Tn7 vector and expressed under the *lac* promoter, led to a small repression of *mfsA* basal levels (0.8 ± 0.1-fold, Fig 3). The degree of repression of *mfsA* by SoxR was fairly low, suggesting that reduced SoxR only has minor role in regulating the basal level of *mfsA*. The major difference between *mfsA* expression in the *soxR* mutant and K279a wild-type was the inability of superoxide generators to induce *mfsA* expression in the mutant. The superoxide generator-induced expression of *mfsA* could be recovered in the complemented *soxR* mutant (Δ*soxR*::*soxR*). The results clearly indicate that oxidized SoxR acts as transcription activator of *mfsA* expression.

**Fig 4:**
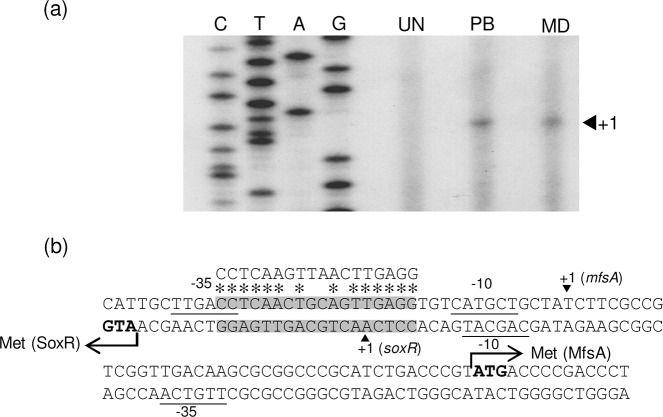
Characterization of the *mfsA*-*soxR* promoter. (a) Primer extension experiments were performed using RNA isolated from uninduced (UN), plumbagin-induced (PB) or menadione-induced (MD) cultures of *S*. *maltophilia* K279a using the ^32^P-labeled BT2833 primer. Extension products were sized on sequencing gels next to DNA sequence ladders (G, A, T, and C) that were generated using the same labeled primer, BT2833. The arrowhead indicates the putative transcription start site (position +1). (b) The nucleotide sequences of both strands of the *mfsA-soxR* promoter region are shown. Upper and lower strands represent the *mfsA* and *soxR* promoters, respectively. The putative -35 and -10 elements are underlined, and the start codon (ATG) is in bold type. The consensus sequence of the *E*. *coli* SoxR binding site is aligned above the sequence line in corresponding letters (gray box), and the homologous nucleotides are marked by asterisks. The arrowhead indicates the putative transcriptional start site (+1).

SoxR mediates gene regulation as a transcriptional regulator through binding to the SoxR binding site of target gene promoters. This feature is conserved in all SoxRs that have been studied in enterobacteria, in which SoxR regulates a single gene, *soxS* (encoding transcriptional activator), or non-enteric bacteria, where SoxR directly controls genes in its regulon [15,17,18,20,21,32]. The promoter of *mfsA* was then experimentally determined. Primer extension experiments were conducted to map the 5’ end of *mfsA* transcripts. Total RNA samples were prepared from uninduced and paraquat-induced cultures, and primer extension was performed using the ^32^P-labeled BT2833 primer. A primer extension product of 53-bp that corresponded to a transcription start site (+1) at a T residue located 39 nucleotides upstream of the translational start codon (ATG) was observed (Fig 4A and 4B). The putative -35 and -10 promoter motifs were identified as TTGACC and CATGCT, respectively, and were separated by 19 bp. The SoxR binding site sequence and its location in relation to the target promoter motifs are remarkably well conserved in diverse groups of Gram-negative bacteria [14,15,17,18,19,20,21] [32]. Analysis of the nucleotide sequence in the vicinity of the *mfsA* promoter revealed an inverted repeat sequence, 5’ACCTCAACTGCAGTTGAGGT3’, that shared high identity (14 of 18 nucleotides) with the consensus sequence for an *E*. *coli* SoxR box (5’CCTCAAGTTAACTTGAGG) [14]. The SoxR box is normally located in the intervening sequence between the -35 and -10 promoter motifs with a slightly imperfect spacer length. Binding of oxidized SoxR to the box leads to alteration in the DNA structure and subsequent activation of transcription of the target gene by RNA polymerases [12,15,18,32]. The *mfsA* promoter architecture fits well with a typical SoxR-regulated promoter, which has an imperfect spacer length and the SoxR box between the -35 and -10 regions.

**Fig 5:**
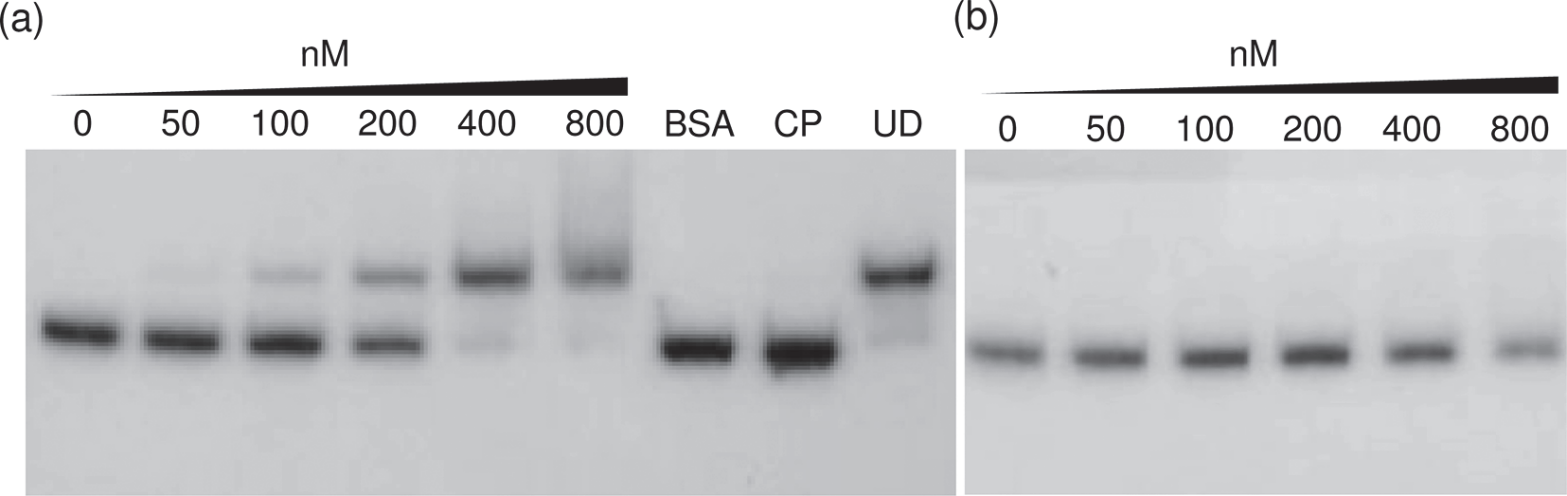
Electrophoretic mobility shift assay. Gel shift experiments were carried out using increasing concentrations of purified reduced SoxR protein (0–800 nM) and a ^32^P-labeled, 258-bp wild-type (a) or mutagenized (b) *mfsA-soxR* promoter fragment. BSA represents an unrelated protein (1 mM BSA). CP and UD signify the cold probe (100 ng unlabeled promoter fragment) and unrelated DNA (1 μg of pUC18 plasmid), respectively, that were added to the binding reaction mixture containing 800 nM SoxR. F and B represent the free and bound probes, respectively.

Gel electrophoretic mobility shift assays with purified *S*. *maltophilia* SoxR and a *mfsA* promoter fragment was performed to test the ability of the transcription regulator to bind specifically to a putative *mfsA* promoter region. SoxR was purified as described in the Methods section. The results in Fig 5 illustrate that purified SoxR was able to bind the *mfsA* promoter sequence. The specificity of SoxR binding was revealed by the ability of unlabeled *mfsA* promoter sequence (cold probe, CP) to compete and prevent the binding between the purified SoxR protein and labeled probe, while an excess amount of unrelated, unlabeled DNA (pUC18, UD) did not compete with the labeled probe in the SoxR-*mfsA* promoter complex (Fig 5A). Additionally, excess of the unrelated protein bovine serum albumin (BSA) was unable to bind the labeled probe. To examine the role of the putative SoxR binding sequences that span the -35 and -10 motifs of the *mfsA* promoter, site-directed mutagenesis was performed to convert the putative SoxR box, 5’CCTCAACTGCAGTTGAGG3’, to 5’AAGACCAGTACTGTGAGG3’, and gel mobility shift experiments were repeated using the mutagenized *mfsA* promoter as a probe. As illustrated in Fig 5B, no binding complexes were detected at any of the SoxR concentrations that were tested, indicating that mutation of the putative SoxR binding site severely affected the binding ability of purified SoxR to the *mfsA* promoter. Thus, the putative SoxR binding site on the *mfsA* promoter is required for SoxR-mediated regulation of *mfsA*. Generally, SoxR in Apo-, reduced, or oxidized form binds to the target promoter sequence with a similar affinity. However, only the oxidized SoxR can activate the transcription of the regulated genes [33,34].

Examination of the *mfsA* promoter/translation initiation site revealed an atypical arrangement. The transcription start site is located 42 bp from the translation initiation site; thus, the *mfsA* mRNA contains a relatively long 5’ untranslated region (UTR). The role of this 5’ UTR in the translational control of *mfsA* is unknown. Furthermore, *mfsA* has a poor ribosome-binding site that precedes the translation initiation codon, ATG, which suggests that the *mfsA* mRNA could be poorly translated.

### *soxR* is autoregulated but constitutively expressed

**Fig 6:**
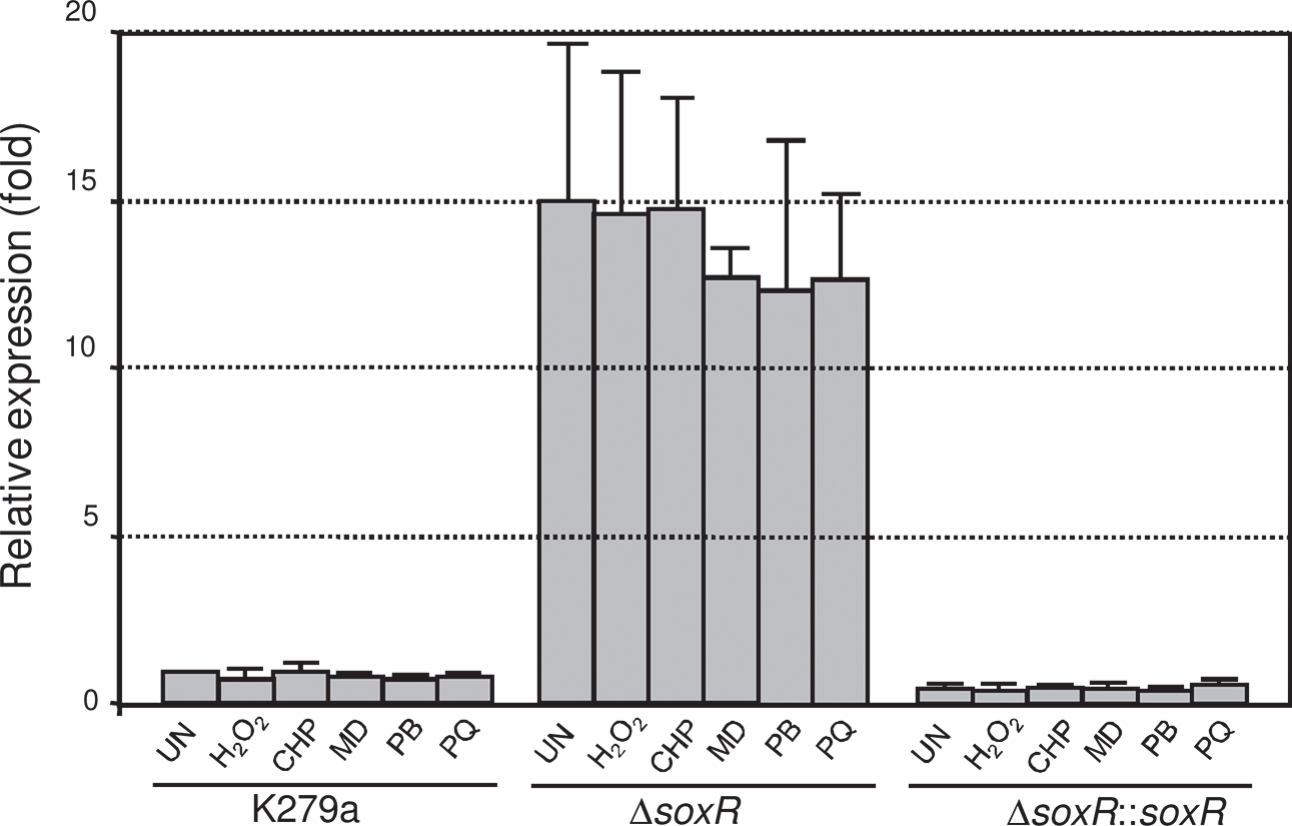
Expression analysis of *soxR*. The expression levels of *soxR* in *S*. *maltophilia* K279a wild-type, the Δ*soxR* mutant and the complemented strain (Δ*soxR*::*soxR*) cultivated under uninduced (UN) conditions and induced with 100 μM H_2_O_2_, 50 μM cumene hydroperoxide (CHP), 200 μM menadione (MD), 100 μM plumbagin (PB) or 100 μM paraquat (PQ) were determined using qRT-PCR. The 16S rRNA gene was used as the normalizing gene. Relative expression was expressed as the fold expression over the level in uninduced K279a.

SoxR typically regulates its own expression, and two types of *soxR* gene expression patterns have been reported. In *E*. *coli* as well as xanthomonads, reduced and oxidized forms of SoxR bind to their promoters and repress their own expression; therefore, *soxR* is constitutively expressed at low levels [14,18]. However, in some bacteria, the expression of *soxR* is inducible upon exposure to redox cycling drugs [15,16]. In *S*. *maltophilia*, *soxR* is located divergently to *mfsA* and shares the same promoter region; and the putative SoxR box is situated 7 bp upstream of the ATG initiation codon (Fig 4). This structure suggests that *soxR* expression is auto-regulated. The expression level of *soxR* was determined in the K279a wild-type, a Δ*soxR* mutant and a Δ*soxR* complemented strain (Δ*soxR*::*soxR*). Total RNA was extracted from exponential-phase cultures that were induced with oxidants. qRT-PCR was conducted using BT2787 and BT3713, and the results are shown in Fig 6. *soxR* was constitutively expressed, and exposure of K279a cultures to redox cycling drugs (menadione, plumbagin and paraquat) and peroxides (H_2_O_2_ and cumene hydroperoxide) failed to induce high-level transcription of *soxR*. A lack of functional SoxR in the Δ*soxR* mutant increased its expression by 15.0 ± 4.6-fold relative to the wild-type level (Fig 6), and challenge with oxidants caused no significant alteration in the *soxR* expression levels. The constitutively high expression of *soxR* in the Δ*soxR* mutant was abolished in the complemented Δ*soxR* strain. Thus, the expression pattern of *soxR* together with the gel mobility shift results in which purified SoxR bound to a putative SoxR box suggest that SoxR in its reduced and oxidized forms acts as a transcriptional repressor. Because the promoter architecture of its target genes is critical for SoxR function, the *soxR* promoter was characterized. We attempted to map the putative transcriptional start site (+1) of *soxR* using primer extension. However, no extension product was detected. Therefore, 5’RACE was employed to determine the 5’ end of the *soxR* transcript. The RACE-PCR products were cloned and sequenced, and the majority of the sequences from 7 independent clones mapped the 5’ end of the *soxR* transcript to an A residue located 21 bp upstream of its ATG codon (Fig 4B). The putative -35 and -10 elements were identified as TTGTCA and CAGCAT, respectively, and they were separated by 17 bp (Fig 4B). The putative SoxR binding site was positioned at -4 to +14 of the *soxR* promoter. The structure of the *soxR* promoter and the SoxR box fit well with the observed expression studies of *soxR* expression (Fig 6). The binding of either reduced or oxidized SoxR to the SoxR box interferes with RNA polymerase binding to the promoter, leading to transcription repression.

### SoxR differentially controls *mfsA* and *soxR* gene expression

*E*. *coli* SoxR is a homodimer protein containing a pair of [2Fe-2S] clusters. This feature is believed to be completely conserved in other SoxRs from various bacteria, including *S*. *maltophilia*, because the [2Fe-2S] anchor motif is highly conserved [20] (data not shown). Altogether, our results suggest that, under physiological conditions, reduced SoxR binds to the single SoxR binding site that is located on the *mfsA-soxR* promoter and represses the expression of *mfsA* and *soxR*. Expression analysis of *mfsA* and *soxR* in the Δ*soxR* mutant indicates that SoxR represses its own expression to a greater extent than that of *mfsA*. The promoter architecture of *mfsA*, in which the SoxR-binding site is located between the -35 and -10 promoter elements with a 19-bp spacer, is similar to typical, SoxR-regulated promoters, where binding of reduced SoxR impairs the interaction between RNA polymerase (RNAP) and promoters: binding of oxidized SoxR distorts the promoter to a configuration that facilitates initiation of transcription by RNAP, thereby activating transcription [14,35]. However, binding of reduced and oxidized SoxR to the sequence covering the +1 site that corresponds to the *soxR* promoter occludes its own transcription by interfering with RNAP binding [14]. The scenario of SoxR-mediated activation and repression of two genes from a single binding site is analogous to that reported in *E*. *coli*, in which *soxR* and a divergently transcribed *soxS* share a promoter sequence that contains a single SoxR binding site. Therefore, oxidized SoxR functions as activator for *soxS* transcription and as an autorepressor [14]. A similar scenario was observed for *S*. *maltophilia* SoxR where it acts as an autorepressor, but as an activator for *mfsA* expression, which is dependent on oxidized SoxR to increase its low basal level of expression.

## Conclusion

We show here that MfsA (Smlt1083), which belongs to a DHA14 subfamily of MFS efflux pumps, plays an important role in mediating resistance to paraquat. In vitro and in vivo evidence indicate that *mfsA* expression is under the control of SoxR, a transcriptional regulator that is activated by exposure to redox cycling drugs including paraquat, plumbagin and menadione. Paraquat is among the redox cycling drugs that potently induce *mfsA* transcription, most likely through oxidation of SoxR [2Fe-2S]. Paraquat is also recognized as a herbicide that is widely used in agricultural fields to control unneeded weeds. Because the usual habitat of *S*. *maltophilia* is soil, the *soxR-mfsA* system would directly benefit the survival of this bacterium in paraquat-contaminated environments. In addition, it is likely that plumbagin and menadione are gratuitous inducers of SoxR with regard to *mfsA* since MfsA does not increase resistance to these oxidative agents. In several bacteria, superoxide dismutases are involved in redox-cycling drug resistance by catalyzing dismutation of superoxide anion. There are reports reveal that SoxR plays a role in *sod* regulation [15,32,36]. *S*. *maltophilia* also contains *sod* genes, thus the contribution of SoxR to regulation of *sod* gene expression and the role of *sod* genes in conferring to redox cycling drug resistance is being investigated.

## Methods

### Bacterial growth conditions

The *Stenotrophomonas maltophilia* wild-type K279a [24] and mutant strains were grown aerobically in LB medium at 35°C with continuous shaking at 150 rpm. Overnight cultures were inoculated into fresh LB medium to give an optical density at 600 nm (OD_600_) of approximately 0.1. Exponential phase (OD_600_ of 0.5, after 4 h of growth) cells were used in all experiments, as indicated. Bacterial strains and plasmids used in this study were listed in Table 1.

**Table 1:** Bacterial strains and plasmids used in this study.

Strain or Plasmid	Relevant characteristic(s)	Source
***S*. *maltophilia***		
K279a	Wild-type strain	[24]
K279a::Tn7T	K279a transposed with pUC18-mini-Tn7T-LAC	This study
Δ*mfsA*	K279a Δ*mfsA* mutant	This study
Δ*mfsA::mfsA*	Δ*mfsA* mutant transposed with pTn7T*mfsA*	This study
Δ*soxR*	K279a Δ*soxR* mutant	This study
Δ*soxR::soxR*	Δ*soxR* mutant transposed with pTn7T*soxR*	This study
**Plasmid**		
pUC18-mini-Tn7T-LAC	pUC18 containing mini-Tn7T::P*_lac_* site, Ap^r^, Gm^r^	[30]
pTn7T*mfsA*	pUC18-mini-Tn7T-LAC containing mfsA	This study
pTn7T*soxR*	pUC18-mini-Tn7T-LAC containing soxR	This study
pTNS2	Helper plasmid encoding the TnsABCD transposase, Ap^r^	[30]
pDrivePmfsA	pDrive cloning vector (Qiagen) containing *mfsA-soxR* promoter sequence, Ap^r^, Km^r^	This study
pGemP*mfsA	pGemT-easy cloning vector (Promega) containing mutagenized *mfsA-soxR* promoter sequence, Ap^r^	This study
pETsoxR_SM_	pET-blue2 vector (Novagen) containing soxR for high expression of *SoxR*, Ap^r^	This study

### Molecular biology techniques

General molecular genetics techniques, including bacterial genomic DNA preparation, plasmid preparation, PCR, gene cloning, transformation into *E*. *coli* and agarose electrophoresis were performed using standard protocols [37]. *S*. *maltophilia* was transformed by electroporation using a 0.2-cm electrode gap cuvette with a Gene Pulser electroporator (BioRad) set at 2.5 kV, 200 Ω and 25 μF.

### Determination of resistance levels to oxidants

The resistance levels against oxidants were determined using a plate sensitivity assay, as previously described [38]. Briefly, exponential-phase cells were 10-fold serially diluted in fresh LB medium before 10 μl of each dilution was spotted onto LB plates containing 400 μM paraquat, 3 mM menadione or 400 μM plumbagin. The plates were incubated at 30°C for 18 h before the numbers of colony forming units (CFUs) were scored. The resistance level to oxidant was expressed as the percent survival, which was defined as the percentage of CFUs on the plates containing oxidant over the CFUs on control plates without oxidant.

### Construction of *mfsA* and *soxR* mutants

The unmarked gene deletion of *mfsA* was constructed using the allelic exchange method with the *cre-lox* marker recycling system [39]. The 3.6-kb DNA fragment spanning the *mfsA* coding sequence plus the sequences flanking both *mfsA* termini was PCR amplified from *S*. *maltophilia* K279a genomic DNA using primers BT2897 and BT2898 (Table 2) before being cloned into pDrive (Qiagen). The recombinant plasmid was digested with NcoI and SacII to excise the 928-bp sequence of the *mfsA* coding region prior to ligation with a NcoI-SacII-cut *loxP*-flanked Gm^r^ cassette (from pUC18Gm [40]), which generated pDrive-ΔmfsA::Gm. This suicide plasmid was introduced into *S*. *maltophilia* K279a using electroporation. Putative mutants generated from a double-crossover event were selected from colonies with the Gm^r^ phenotype and negative PCR results using primers located on the deleted sequence of *mfsA*. The unmarked Δ*mfsA* mutant was generated using Cre-mediated *loxP* recombination to excise the Gm^r^ marker and was confirmed using PCR with primers flanking the deletion sequence.

**Table 2:** List of oligonucleotide primers.

Primer	Sequence (5’ → 3’)
BT2643	CGCGACCACGTTGCCACG
BT2644	AGCTACCCGCAGCACATG
BT2646	CGATGCAGCCGGTGAGCTC
BT2781	GCCCGCACAAGCGGTGGAG
BT2782	ACGTCATCCCCACCTTCCT
BT2787	GTTGAGGTCAAGCAATGGT
BT2788	TTCGCGGTTCACTCCCAGC
BT2832	GCGGATCACCGCCAGCCG
BT2833	GAGGGTCGGGGTCATACG
BT2835	GCGGCGCTACACCCGTCG
BT3246	GCCATGGTGTCCCAGGAACTGA
BT3247	TCTCGAGTCACTCCCAGCGCATC
BT3713	CCTTGCGCTGCTAGAAGTG
BT3766	ACGACAGCCTGCTGGAGAA
BT3767	CACAGCATAACTGGACTGATT
BT3817	GTAGCAGCAGGGGCAGATA
BT3818	GGGATGTGCTTGGCGAAC
BT3819	GGATGTACTTGCCGACCATC
BT4081	AAGACCAGTACTGTGAGGTGTCATGCTGCT
BT4082	CAGTACTGGTCTTTCAAGCAATGGTGTCCC

The Δ*soxR* mutant was constructed. The 978-bp downstream region of *soxR* was PCR amplified from K279a genomic DNA using primers BT3818 and BT3819 (Table 2). The PCR product was cloned into pGem-T (Promega, USA) before the NcoI-NotI fragment was subcloned into pUC18Gm, giving pΔSoxR-GmDown. Then, the 1,155-bp upstream sequence of *soxR* was amplified from K279a genomic DNA using primers BT3817 and BT3713 (Table 2) and cloned into pGem-T prior to subcloning the SacI-ApaI fragment into pΔSoxR-GmDown, which generated pΔSoxR-UpGmDown. This plasmid was introduced into K279a, and the Δ*soxR* mutant was selected from colonies with carbenicillin-susceptible (Cb^s^) and Gm^r^ phenotypes. Excision of the Gm^r^ cassette from the Δ*soxR* mutant was attained through Cre-*loxP* recombination, and the unmarked Δ*soxR* mutant was confirmed using PCR with two primers that flanked the deletion site.

### **Construction of the** Δ***mfsA* mutant complemented strain (**Δ***mfsA*::*mfsA*)**

A single copy of *mfsA* was inserted into the chromosome of the *S*. *maltophilia mfsA* mutant using a Mini-Tn7 transposon vector. A DNA sequence spanning the full-length *mfsA* gene and its putative promoter was PCR amplified using the BT3713 and BT2835 primers (see Table 2), and the PCR product was cloned into pGemT-easy (Promega). The inserted DNA was sequenced to assure that no mutation had occurred before the ApaI-SacI fragment containing *mfsA* was cloned into pUC18 mini-Tn7T-LAC [30] that was cut with the same restriction enzymes. The resulting plasmid pTn7T*mfsA* and pTNS2 [30], which encoded the TnsABCD proteins that are required for *att*Tn7 site-specific transposition, were transferred into the *mfsA* mutant. The transformants were selected on plates containing gentamicin. Integration of the mini-Tn7 transposon generally takes place at *att*Tn7, which is located downstream of *glmS*, which encodes glucosamine-fructose-6-phosphate aminotransferase, an enzyme that catalyzes the formation of glucosamine 6-phosphate. *S*. *maltophilia* K279a contains a single *glmS* (*smlt4099*) gene and a sequence that shares high identity with the *att*Tn7-associated sequence [41] was identified near the 3’ end of the putative *glmS* gene. Integration of the mini-Tn7 containing *mfsA* into the mutant genome was assured by PCR amplification of the genomic DNA using a primer pair; one that annealed to the *glmS* gene (BT3766) and another that annealed to Tn7R (BT3767), as previously described for *Xanthomonas campestris* [41].

### **Construction of the** Δ***soxR* mutant complemented strain (**Δ***soxR*::*soxR*)**

Single copy complementation of the Δ*soxR* mutant was achieved using a mini-Tn7 transposon vector. The full-length *soxR* gene was PCR amplified using primers BT2787 and BT2788 and K279a genomic DNA as template. The blunt-end PCR product (435 bp) was cloned into pUC18-mini-Tn7T-LAC [30] at the EcoICRI site, the resulting plasmid pTn7T*soxR* and pTNS2 were transferred into the Δ*soxR* mutant, and the transformants were selected and confirmed, as described for the construction of the Δ*mfsA* mutant complemented strains.

### Real-time reverse transcription PCR (qRT-PCR)

Exponential *S*. *maltophilia* cultures were induced with sub-lethal concentrations of oxidants (100 μM hydrogen peroxide [H_2_O_2_], 50 μM cumene hydroperoxide [CHP], 200 μM menadione [MD], 100 μM plumbagin [PB], 100 μM paraquat [PQ]) for 15 min before harvesting for total RNA preparation. qRT-PCR was performed as previously described [42]. The reverse transcription reaction was conducted using 5 μg of total RNA, the RevertAid M-MuLV Reverse transcriptase kit (Fermentas) and random hexamers, according to the manufacturer’s recommendation. The specific primer pairs that were used included BT2787-BT3713 for *soxR*, BT2643-BT2644 for *mfsA* and BT2781-BT2782 for the 16S rRNA gene, which was used as the normalizing gene. The BT2787 and BT3713 primers are located within the 5’ region of the *soxR* transcript and upstream of the deletion site of the Δ*soxR* mutant. qPCR was performed using 20 ng of cDNA, a specific primer pair and SYBR green PCR Master Mix (Applied Biosystems). The PCRs were run on an Applied Biosystems StepOne Plus under the following conditions: denaturation at 95 °C for 30 s, annealing at 58 °C for 45 s and extension at 72 °C for 45 s, for 40 cycles. Relative expression was calculated using the STEPONE software v2.1 and expressed as fold expression over the level of K279a wild-type that was cultivated under uninduced conditions. Experiments were independently repeated three times.

### Purification of the *S*. *maltophilia* SoxR protein

The untagged SoxR protein was highly expressed using the pET-blue2 expression vector and *E*. *coli* system (Novagen). The *soxR* full-length gene was PCR amplified from K279a chromosomal DNA using primers BT3246 and BT3247, and the PCR products were digested with NcoI and XhoI and cloned into the pET-Blue-2 that was cut with the same enzymes, which generated pETsoxR_SM_. Purification of untagged SoxR was conducted as previously described [15]. Essentially, exponential-phase cultures of *E*. *coli* BL21 (DE3)/pLacI harboring pETsoxR_SM_ were induced with 1 mM isopropyl-β-D-thiogalactopyranoside (IPTG) for 2 h. SoxR was then purified aerobically using a Whatman P-11 phosphocellulose ion exchange column. Oxidized SoxR protein was eluted with a linear gradient of 0.2 to 1.0 M KCl, and the purity of the SoxR protein was estimated by SDS-PAGE analysis.

### Cloning of the *mfsA* promoter and site-directed mutagenesis

A 258-bp DNA fragment spanning the putative *mfsA-soxR* promoter was PCR amplified from K279a genomic DNA using primers BT2832 and BT2833 and subsequently cloned into the pDrive vector (Qiagen, Germany), which generated pDrivePmfsA. Two-step, PCR-based, site-directed mutagenesis [18] was performed to change the putative SoxR binding site sequence (5’CCTCAACTGCAGTTGAGG3’) to 5’AAGACCAGTACTGTGAGG3’ using two mutagenic primers, BT4081 and BT4082 (Table 2), and pDrivePmfsA as a DNA template. The PCR product was cloned into pGemT-easy (Promega, USA), generating pGemP*mfsA. To ensure that no unexpected mutation occurred, the nucleotide sequences of the insert DNA in pDrivePmfsA and pGemP*mfsA were determined.

### Primer extension

Total RNA was extracted from exponential-phase cultures grown under induction with 200 μM menadione or 100 μM plumbagin or uninduced conditions. The ^32^P-labeled primer BT2833 (Table 2) was mixed with 10 μg of DNase I-treated RNA, and reverse transcription was performed using Superscript III reverse transcriptase (Invitrogen) at 60°C for 60 min. Extension products were sized on sequencing gels (9% polyacrylamide–7M urea) next to dideoxy sequencing ladders that were generated using a PCR sequencing kit (Applied Biosystems) with labeled BT2833 and pDrivePmfsA plasmid containing a putative *mfsA* promoter fragment as templates.

### Electrophoretic mobility shift assay

The putative *mfsA* promoter was amplified using the BT2832 and ^32^P-labeled BT2833 primers and pDrivePmfsA or pGemP*mfsA (for the mutagenized promoter) as a DNA template. Electrophoretic mobility shift assays were then performed by adding 20 ng of ^32^P-labeled, 258-bp *mfsA* promoter to a 25-μl reaction mixture (12 mM HEPES-NaOH buffer [pH 7.9], 10 mM KCl, 1 mM dithiothreitol [DTT], 12% glycerol, 50 μg ml^-1^ bovine serum albumin, 5 μg ml^-1^ calf thymus DNA, 0.5 mg ml^-1^ poly(dI/dC)] containing increasing concentrations of purified, oxidized SoxR (0 to 800 nM). The binding reactions were incubated at 25°C for 30 min, and the protein-DNA complexes were analyzed by electrophoresis on a 7% native polyacrylamide gel in 0.5×Tris-borate-EDTA buffer at 4°C.

### 5’ rapid amplification of cDNA ends (RACE)

5’ RACE was performed using a 5’/3’ RACE kit per the manufacturer’s recommendation (Roche, Germany). Essentially, DNase I-treated total RNA was reversed transcribed using a specific primer BT2646 (SP1). The first-strand DNA (cDNA) was purified, and poly(A) was added to the 5’-terminus of the cDNA using terminal transferase enzyme. Next, poly(A)-tailed cDNA was PCR amplified using the specific BT3713 (SP2) primer and an anchored oligo(dT) primer. The purified PCR product was cloned into the pGemT vector, and the +1 site was identified from the DNA sequences.
